# First-generation vs second-generation antipsychotic drugs: The ongoing saga

**DOI:** 10.4103/0019-5545.58904

**Published:** 2010

**Authors:** Sumeet Gupta

**Affiliations:** West Pak Hospital, Darlington, UK. E-mail: sumeetgupta_2000@yahoo.com

Sir,

I have read the letter to editor by Dasgupta and Dasgupta with interest.[1] In this year, we have some more publications shedding more light in this area. It is a very intricate area and one should be cautious in interpreting the evidence. Firstly, we have evidences from both efficacy vs effectiveness trial like CATIE (Clinical antipsychotic trials in intervention effectiveness), and CUTLASS (Cost utility of the latest antipsychotics in severe schizophrenia). Effectiveness trials or practical clinical trials are more informative and can easily be applied to practice. Secondly, second-generation antipsychotic drugs (or atypical antipsychotics) are not a homogenous category, they differ both in terms of effects and side effects; hence, when comparing to first-generation drugs, we should be cautious about generalization of the findings.[2] The superiority of clozapine, especially in treatment-resistant patients, has been established. The position of other second-generation antipsychotic drugs, in comparison to first-generation antipsychotic drugs, remains uncertain.

Leucht *et al*. recently published a meta-analysis of 150 double blind clinical trials, involving 21,533 patients.[3] Four atypical antipsychotic drugs were found to be better than first-generation antipsychotic drugs for overall efficacy (both positive and negative symptoms). Among them, the effect size was largest for clozapine −0.52 (95% CI −0.75 to −0.29, *P* < 0.0001), which was not surprising. Clozapine was followed by amisulpride −0.31(−0.44 to −0.19, *P* < 0.001), olanzapine −0.28 (−0.38 to 0.18, *P* < 0.0001) and lastly risperidone −0.18 (−0.22 to −0.05, *P* = 0.002). The other second-generation antipsychotics (aripiprazole, quetiapine, zotepine, ziprasidone, sertindole) were not more efficacious than first-generation drugs, even on negative symptoms. Extrapyramidal (EP) symptoms, as expected, were more on first-generation antipsychotic drugs. On the other hand, second-generation antipsychotics drugs (except ziprasidone and aripiprazole) induced more weight gain. Similarly, certain drugs were better for depressive symptoms (amisulpride, clozapine, olanzapine, aripiprazole, and quetiapine). They also showed variable changes in overall efficacy of the individual drug when they compared the efficacy in industry-sponsored or non-sponsored trials.

In March 2009, NICE updated the previous guideline issued in 2002.[4] While in the previous guideline, they had recommended first-line use of atypical antipsychotic drug (second generation). In the current update, due to recent evidence casting doubts about alleged superiority of second-generation antipsychotic drugs, NICE have suggested that selection of the antipsychotic drug should be based on benefit and side-effect profile (EP symptoms vs metabolic side effects). NICE no longer recommends second-generation antipsychotic drugs as a first-line treatment.

The artificial classification of the antipsychotic drugs has caused more harm than benefit. The dichotomy of typical and atypical antipsychotic drugs has been abandoned. The current splitting, first generation and second generation, is also not satisfactory as second generation implies that these drugs are advanced and better than the previous ones. What are the implications of these findings? We should not restrict ourselves to second-generation antipsychotic drugs. In some patients, first-generation antipsychotic drugs may be more effective and better tolerated than the second-generation antipsychotic drugs. The long-term studies with global outcome measures (quality of life, functioning) rather than only symptomatic outcomes will help in understanding the risks and benefits of individual antipsychotic drug.
